# Achieving Passive Localization with Traffic Light Schedules in Urban Road Sensor Networks

**DOI:** 10.3390/s16101662

**Published:** 2016-10-10

**Authors:** Qiang Niu, Xu Yang, Shouwan Gao, Pengpeng Chen, Shibing Chan

**Affiliations:** School of Computer Science and Technology, China University of Mining and Technology, Xuzhou 221116, China; niuq@cumt.edu.cn (Q.N.); yamgxu@cumt.edu.cn (X.Y.); gaoshouwan@cumt.edu.cn (S.G.); 08133468@cumt.edu.cn (S.C.)

**Keywords:** wireless sensor network, localization, traffic lights, time stamps

## Abstract

Localization is crucial for the monitoring applications of cities, such as road monitoring, environment surveillance, vehicle tracking, etc. In urban road sensor networks, sensors are often sparely deployed due to the hardware cost. Under this sparse deployment, sensors cannot communicate with each other via ranging hardware or one-hop connectivity, rendering the existing localization solutions ineffective. To address this issue, this paper proposes a novel Traffic Lights Schedule-based localization algorithm (TLS), which is built on the fact that vehicles move through the intersection with a known traffic light schedule. We can first obtain the law by binary vehicle detection time stamps and describe the law as a matrix, called a detection matrix. At the same time, we can also use the known traffic light information to construct the matrices, which can be formed as a collection called a known matrix collection. The detection matrix is then matched in the known matrix collection for identifying where sensors are located on urban roads. We evaluate our algorithm by extensive simulation. The results show that the localization accuracy of intersection sensors can reach more than 90%. In addition, we compare it with a state-of-the-art algorithm and prove that it has a wider operational region.

## 1. Introduction

Wireless sensor networks are usually used for monitoring activities in the city. The localization of sensors is crucial for monitoring activities because monitoring messages often make no sense without the location information.

Localization schemes have been studied extensively in wireless sensor network over the past few years. There are basically two types of methods: range-based localization schemes (e.g., Received Signal Strength Indicator (RSSI) [1], Time of Arrival (TOA) [2], Time Differences of Arrival (TDOA) [3] and Angle of Arrival (AOA) [4]) or range-free localization schemes (centroid [5], robust quads [6], Secure Range-Independent Localization (SeRLoc) [7] and Approximate Point-In-Triangulation (APIT) [8]). Range-based localization schemes are very accurate, but they are not suitable for the large-scale urban road sensor networks, due to some shortcomings (such as extensive system calibration or environment profiling [1], costly for requiring per-node ranging hardware [3]). Rang-free methods localize with simple sensing, such as anchor proximity [5] and wireless connectivity [9], which have a low system cost. However, it sacrifices localization accuracy for urban road sensor networks. Moreover, the sensor deployment is generally very sparse in urban areas. In this scenario, sensors cannot reach each other through either ranging devices (e.g., ultrasound signals can propagate only 20–30 feet) or single-hop RF connectivity. Thus, the existing localization schemes become ineffective.

To address this issue, we put forward a Traffic Lights Localization (TLS) algorithm based on traffic light information to solve the location problem with sparse deployment of sensors on urban roads. This algorithm is built on an observation: controlled by the traffic lights, the time segment of vehicles and no vehicles is a regular cycle on each road. Additionally, some literature [10,11,12], which is related to the urban traffic light scheduling problem, points out that the time segment of vehicles is a regular cycle. Therefore, the challenging research question becomes how to use vehicle detection time stamps to determine the time law, and then, according to the time law, how to find the node nearest to the intersection.

Our main idea is that through statistical analysis of vehicle detection time stamps, we can obtain a road law and express this law as a matrix called a detection matrix. On the other hand, we use known traffic information to construct a matrix called a known matrix that also contains the road law. Then, we find which detection matrix and which known matrix are similar. Through the matrix match, we can know the nodes nearest to the intersection. Some digital maps provide traffic light schedule information at the intersection. Such a kind of digital map has already been commercialized [13]. The latest one is developed by MapMechanics [14]. In addition, the program of setting traffic lights and the traffic public facilities data can be queried in the corresponding traffic department. There are many literature works [15,16,17,18,19,20] about the analysis of big and heterogeneous data generated by a diversity of sources in urban spaces, such as sensors, devices, vehicles, buildings and humans, to tackle the major issues that cities face, e.g., air pollution, increased energy consumption and traffic congestion. As the data of public facilities in the literature are achieved from the relevant departments, so the scheduling information data of the traffic lights can be queried by the relevant departments, and the position of the traffic lights can be queried through Google Maps. Therefore, we know the geographical position of the intersection and locate the intersection nodes. Finally, when some intersection nodes have been located, it is easy to locate other nodes by relying on the map matching method of the Autonomous Passive Localization (APL) algorithm [21].

Specifically, our localization scheme consists of four phases: (1) the construction of the matrix; (2) determining a similar matrix and (3) according to the results of a similar matrix matching, determining the location of the intersection nodes and (4) determining the locations of other nodes by the map matching method of the APL algorithm [21].

Our key contributions in this paper are as follows:

- A novel localization scheme is presented based on the position of public facilities. To our best of knowledge, the TLS algorithm is the first localization scheme that employs the public facilities information.
- The localization is accomplished using only the binary detection of vehicles in an urban road network. Unlike previous approaches, TLS is designed especially for sparse sensor networks where long-distance ranging is difficult. In addition, some practical issues are considered, such as a similar traffic light schedule and some damaged nodes.
- A novel method for calculating the similarity of two matrices is designed. This method can judge the similarity of two matrices even when the row order of a matrix is uncertain.
- The performance of the proposed design is evaluated by extensive simulation studies. The results show that our localization scheme can work well.

The rest of this paper is organized as follows. Section 2 describes related work for the localization in wireless sensor networks. Section 3 describes the problem formulation for our traffic lights Localization. In Section 4, our TLS system design is described. In Section 5, we give practical discussions that can affect our localization scheme in practice. Section 6 evaluates our TLS algorithm in realistic settings and compares it to the APL algorithm. In Section 7, we conclude this paper and anticipate future work.

## 2. Related Work

At present, there are many sensor localization schemes, which can be divided into three categories: (1) range-based localization schemes; (2) range-free localization schemes and (3) event-driven localization schemes.

Range-based localization schemes need costly hardware devices (e.g., ultrasound ranging devices) to accurately calculate the distance between nodes [22,23]. The main idea of these schemes is to use the distance of nodes for sensor localization. The advantage of these schemes is the accuracy rate of localization. Considering that these schemes must work with sensors within a short distance, they are unfit for localization in sparse urban road sensor networks.

Range-free localization schemes are able to locate unknown nodes without actual measurement of the absolute distance between nodes [24,25]. They can obtain the relative position of nodes by other information (e.g., geometric relationship and hop), to estimate the localization of unknown nodes [26,27,28,29,30]. These schemes reduce the demand for hardware and are suitable for large-scale dense deployment of sensor nodes in the network. Thus, these schemes do not work well in sparse urban road sensor networks.

Lately, many event-driven localization schemes have been proposed to simplify the functionality of sensors for localization and to provide high quality localization [31,32,33,34,35]. The key idea of these schemes is to use artificial events for sensor localization that are generated from the event scheduler. Although their effective range can reach hundreds of meters, additional external devices and manual operations are needed to generate artificial events. On the other hand, our localization scheme is a new branch of event-driven localization schemes. Because our localization scheme is based on the natural events of moving vehicles, event delivery is not problematic.

The traffic lights, as one key component in this paper, play a decisive role in urban traffic. Because urban traffic congestion is a common phenomenon at crossroads, reasonably setting up traffic light time is key to solving the problem of traffic congestion. Consequently, there is extensive literature about traffic light information [36,37] that points out that traffic light time changes at constant cycle times. In our TLS, we make full use of the traffic light information to finish localization.

## 3. Problem Formulation

**Figure 1:**
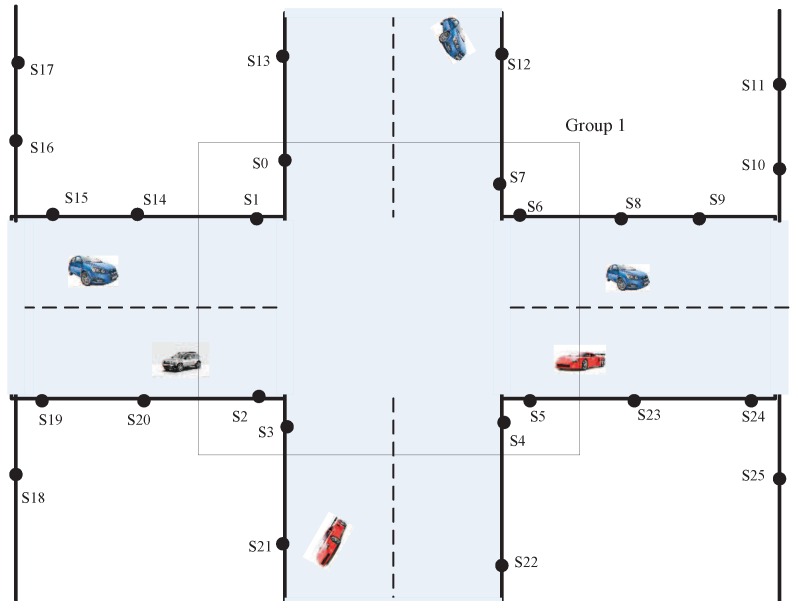
Intersection with wireless sensors.

We consider a network model where sensors are placed at urban road networks. The objective is to locate wireless sensors deployed in road networks only with traffic light information and binary vehicle detection time stamps taken by sensors, as shown in Figure 1. Section 3.1 lists the definitions for TLS, and Section 3.2 lists the assumptions.

### 3.1. Definitions

We define six terms as follows:

1. Intersection node group: We choose eight sensors near the intersection as a group. This sensor group is called the intersection node group. In Figure 1, sensors $S_{0},\;S_{1},\;S_{2},\;S_{3},\;S_{4},\;S_{5},\;S_{6}$ and $S_{7}$ compose an intersection node group.
2. Key nodes: Sensors are placed at intersections. These sensors belong to an intersection node group. In Figure 1, sensors $S_{0},\;S_{1},\;S_{2},\;S_{3},\;S_{4},\;S_{5},\;S_{6}$ and $S_{7}$ are key nodes.
3. Common nodes: Sensors are placed at non-intersections. These sensors do not belong to any intersection node group. In Figure 1, sensors $S_{20},\;S_{21},\;S_{22}$ and $S_{23}$ are common nodes.
4. Detection matrix collection: Analysis of data collected by each intersection node group, which constructs a detection matrix. All of the detection matrices compose the detection matrix collection.
5. Known matrix collection: Using the intersection traffic light information that is obtained from the transport sector, we can construct a known matrix for each intersection. All of the known matrices compose the known matrix collection.
6. TLS server: A computer that performs the localization algorithm with binary vehicle detection time stamps collected from the sensor network.

### 3.2. Assumptions

The localization design of TLS is based on the following assumptions:

- Sensors have simple sensing devices without any costly ranging or GPS devices. Each detection is a tuple $\left(s_{i},g_{i},t_{i}\right)$ consisting of a sensor ID $s_{i}$, intersection node group ID $g_{i}$ and time stamp $t_{i}$. For the common nodes not belonging to any intersection node group, the tuple $\left(s_{i},0,t_{i}\right)$ is sent to the TLS server.
- The traffic light information of the target area is shown on the TLS server. The information consists of traffic time seconds and of a traffic light’s location.
- Sensors deployed in the road on both sides so that each intersection is guaranteed to have one intersection node group.
- Each intersection node group of eight nodes is close to the intersection so that the key nodes and intersection location are basically identical.
- An existing ad hoc network consisting of sensors or a Delay-Tolerant Network (DTN) for wireless sensors aims to deliver vehicle-detection time stamps to the TLS server. For such a DTN, utilizing the Vehicular Ad Hoc Network (VANET) forwarding schemes, such as Vehicle-Assisted Data Delivery(VADD) [13] and Trajectory-Based Data(TBD) [38], to deliver the time stamps to the TLS server, VANETs are constructed by the vehicles, which are data mules [39].
- Sensors are time-synchronized at the millisecond level. We can use the time synchronization protocol in [40] to ensure the time synchronization accuracy in sparse urban road sensor networks; since the time synchronization protocol in [40] is the start-of-the-art time synchronization protocol for spare wireless sensor networks and can ensure the time-synchronized at the millisecond level.

## 4. TLS System Design

### 4.1. System Architecture

**Figure 2:**
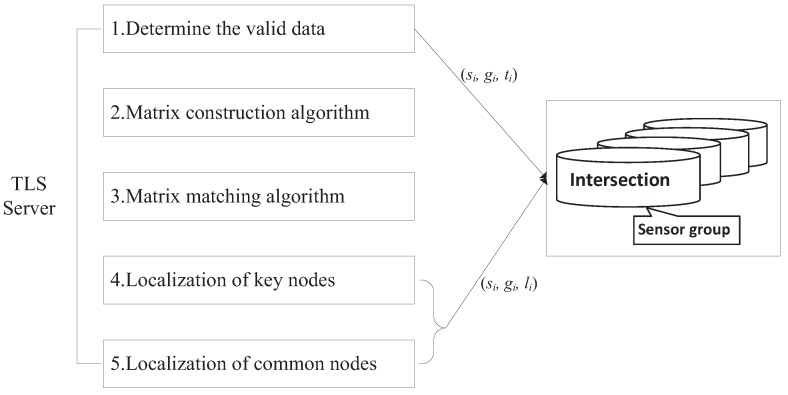
TLS system architecture.

We use an asymmetric architecture for localization, as shown in Figure 2. The sensors register only vehicle-detection time stamps into their local repositories to simplify the functionality of sensors for localization. The TLS server processes the complex computation for localization. Specifically, the localization process includes the following steps, as shown in Figure 2.

- Step 1. After road traffic measurement, sensors send a tuple to the TLS server, i.e., $\left(s_{i},g_{i},t_{i}\right)$, where $s_{i}$ is the sensor ID, $g_{i}$ is the intersection node group ID and $t_{i}$ is a time stamp.
- Step 2. Determining the valid data: The data collected in the heavy traffic time is regarded as valid data, because the time period with cars on the road and the time period without cars is a regular cycle. In the following, a method is designed to screen out the valid data.
- Step 3. By using valid data collected from nodes, the information from each intersection node group can be used to construct a detection matrix. These matrices constitute the detection matrix collection.
- Step 4. We use our algorithm to determine the similar matrix in detection matrix collection and known matrix collection.
- Step 5. Because we already know the geographical location of traffic lights, we can locate the position of the key nodes according to the matrix matching results (obtained from Step 4), and then, we can use the APL algorithm to locate the position of the common nodes.
- Step 6. The TLS server sends each sensor $s_{i}$ its location with a message $\left(s_{i},g_{i},l_{i}\right)$.

### 4.2. Step 2: Determine the Valid Data

#### 4.2.1. Determining the Valid Data Operation

When vehicle traffic is sparse, the time with cars on the road and the time without cars is irregular. However, in heavy traffic times, due to the traffic light control, the time with cars on the road and the time without cars form a regular cycle, and we can use the recurrence to construct a detection matrix. Thus, we need to determine heavy traffic time data, which will be valid times for data to construct a detection matrix. The following introduces a data operation for determining valid data.

**Figure 3:**
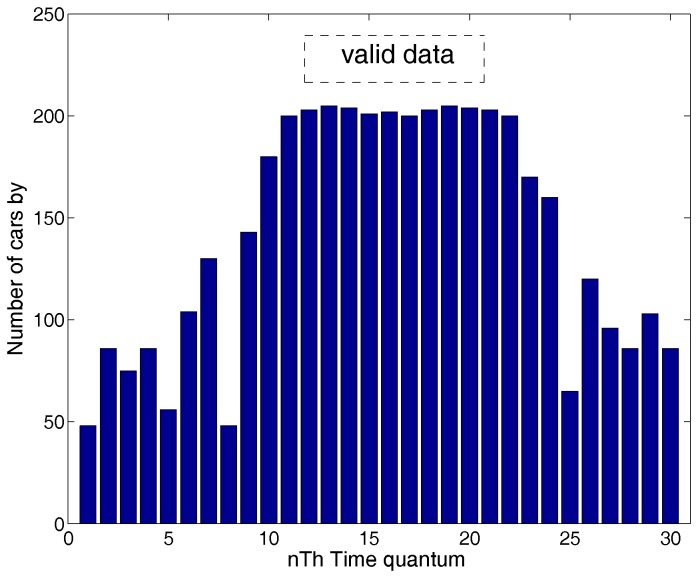
Determine valid data operation.

First, we divide the data into many time segments and calculate the number of vehicle in each time segment [21]. If the number of vehicles is significant and basically remains unchanged in the next time segment, the data of this time segment are seen as valid data. For example, in Figure 3, from the thirteenth time segment to the twenty-first, the value (the number of vehicles) is high and basically remains unchanged; therefore, these data can be seen as valid data. We tested this method in an intersection by using the method of manual counting and found that our method works well. In crowed times, the number of vehicles is at a great value and basically remains unchanged in each time quantum.

#### 4.2.2. Analysis of Determining Valid Data Errors

The counting process of vehicle arrivals can be modeled as a Poisson process in which the number of vehicle arrivals within an hour is *λ*. Note that this modeling is valid for the following two reasons; (1) the Kolmogorov–Smirnov test can accurately approximate the statistics of vehicle interarrival time based on the empirical data for a real roadway into an exponential distribution [41]; and (2) an exponential distribution for the interarrival time is equivalent to a Poisson distribution for the arrival number within a unit time [42]. Let $P_{error}$ be the screening out valid data error probability. Let *N* be the random variable of the number of vehicle arrivals within a time segment. Let *n* be the number of vehicles arriving at sensor $s_{i}$. Let *t* be the size of a time segment. Thus, the determining valid data error probability $P_{error}$ can be computed as follows:

(1) $$\begin{array}{cc} P_{error} & =P[N>n]\\ & =1-P[N\leq n]\\ & =1-\sum_{k=o}^{n}\frac{{\left(\lambda t\right)}^{k}}{k!}\times e^{-\lambda t} \end{array}$$

We compute $P_{error}$ through the simulation with the parameter settings in Table 1. We use the three parameters λ,t and *n* to compute $P_{error}$. The determining valid data error probability is $P_{error}\approx 0$, where λ=600,n=200 and t=1/6 (h). Therefore, because $P_{error}$ is very small, we claim that the locating valid data operation is very accurate.

### 4.3. Step 3: Matrix Construction Algorithm

#### 4.3.1. Detection Matrix Construction Algorithm

**Figure 4:**
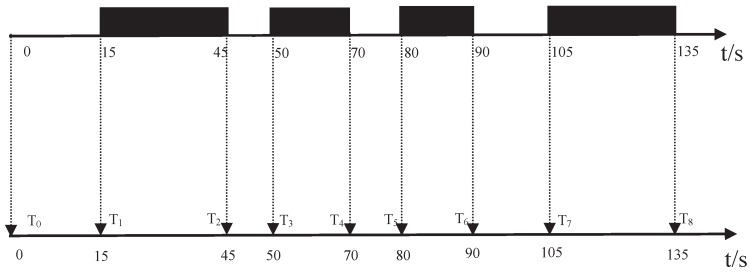
Operation on binary vehicle-detection time stamps.

First, we divide the valid data into two time segments (vehicle passing time period and no vehicle passing time period) and preprocess the vehicle detection data. The adopted method is to screen out the time stamp of the first vehicle arrivals and the time stamp of the last vehicle arrivals in one vehicle passing time period and then form an array *T*. Then, we explain an operation on binary vehicle detection time stamps. The operation is defined as follows:

(2) $$A_{\left[i\right]}=T_{i+1}-T_{i}$$

where $T_{i}$ is the time of the first vehicle arrivals and the time of the last vehicle arrivals in the *i*-th vehicle passing time period and where A[i]∈Afori=1,2..., *A* is an array. According to array *A*, we can obtain the road law and construct the detection matrix. For example, we can obtain an array by the previous operation in Figure 4, which is as follows:

15,30,5,20,10,10,15,30,5,20,10,10,15,30,5,20,10,10

It is easy to see that the array is a regularly-recurring array. That is, taking the largest number 30 as the first number and taking the next five numbers forms a 1 × 6 matrix [30,5,20,10,10,15] that is the matrix analyzed by one sensor. In the same manner, we can obtain the corresponding 1 × 6 matrix of other sensors. Because each intersection node group has eight sensors, we can constitute an 8 × 6 detection matrix for each intersection node group.

#### 4.3.2. Known Matrix Construction Algorithm

We design a vehicle traffic lights simulator using the C# language. This simulator’s main function is to simulate the cases of vehicles passing during heavy traffic time. By importing different information on traffic lights, the system will automatically simulate the intersection passing events of vehicles and record the time stamp. With the simulated data, we also employ the construction algorithm (also used in Section 4.3.1) to construct a known matrix. Thus, each intersection draws a corresponding 8 × 6 known matrix. Additionally, each known matrix corresponds to a tuple $\left(id,L_{i}\right)$, where id is the known matrix ID and $L_{i}$ is the location of the corresponding traffic light.

### 4.4. Step 4: Matrix Matching Algorithms

#### 4.4.1. Calculate the Similarity of the Matrix

In this section, we introduce a method to calculate the similarity of two matrices. For example, there are two matrices, $M_{a}$ and $M_{b}$. Let $a_{\left(i,j\right)}$ be the elements of $M_{a}$, and let $b_{\left(i,j\right)}$ be the elements of $M_{b}$, where *i* is the row index and *j* is the column index. Let $|M_{a}^{r}|$ be the number of rows of $M_{a}$, and let $|M_{a}^{c}|$ be the number of columns of $M_{a}$. We have the formula to calculate the similarity of the matrices as follows.

(3) $$S\left(M_{a},M_{b}\right)=\frac{\sum_{i=1}^{|M_{a}^{r}|}\sum_{j=1}^{|M_{a}^{c}|}C\left(a_{\left(i,j\right)},b_{\left(i,j\right)}\right)}{|M_{a}^{r}\left|\times \right|M_{a}^{c}|}$$

where $C(a_{\left(i,j\right)},b_{\left(i,j\right)})$ aims to calculate the element similarity between $a_{\left(i,j\right)}$ and $b_{\left(i,j\right)}$. Specifically,

(4) $$C\left(a_{\left(i,j\right)},b_{\left(i,j\right)}\right)=\left\{\begin{array}{c} 1,\mathrm{if}\;|a_{\left(i,j\right)}-b_{\left(i,j\right)}|\;<m\\ 0,\mathrm{otherwise} \end{array}\right.$$

where *m* is the matrix element gap threshold. Essentially, we subtract the two corresponding elements in two matrices and fetch the absolute value.

If the result is less than a certain range, we can consider that the two matrix elements are similar. If $S(M_{a},M_{b})>s$ (the matrix similarity threshold), we consider these two matrices similar. In Section 6, the matrix element gap threshold *m* is determined via simulations to be three, and the matrix similarity threshold *s* is determined to be 0.8. We test these two thresholds by many simulation experiments in Section 6.

#### 4.4.2. Row Uncertain Matrix Matching Algorithm

Each group of eight sensors deployed in sequence is uncertain, leading to the matrix’s row order being uncertain. In the same crossroads, the detection matrix obtained by detecting data and the known matrix constructed by simulating the intersection must be similar matrices. However, the basic algorithm from Section 4.4.1 cannot judge whether the two matrices are similar because the row order of the matrix is uncertain. Next, we will introduce two types of algorithms to judge the similarity of two matrices if the row order of a matrix is uncertain.

##### *(a)* *Row Sum Sorting Method (RSS)*

**Figure 5:**
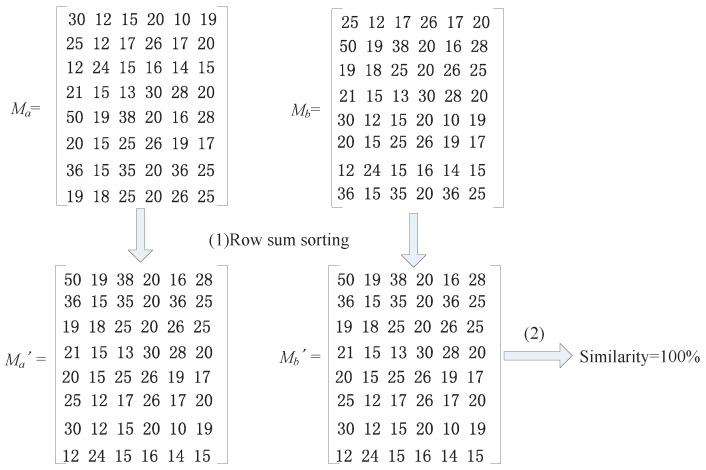
Example for the Row Sum Sorting (RSS) method.

To determine whether two matrices are similar, they should be first sorted, then summed by rows, and after that, the bubble sort method can be used to sort the rows of the matrix. Two matrices that are already sorted can be determined to be similar or not by using the method mentioned in Section 4.4.1. For example, we are going to determine whether two 8 × 6 matrices are similar. As shown in Figure 5, there are two 8 × 6 matrices called $M_{a}$ and $M_{b}$. If we directly use the basic arithmetic of the similarity of two matrices, the similarity of the two matrices would only be 25%. However, if we use the modification of sorting and summing first, we will obtain two intermediate matrices, which can be called $M_{a}^{\prime }$ and $M_{b}^{\prime }$. Using the basic arithmetic to calculate the similarity of the two intermediate matrices obviously yields 100%, which is the true similarity of matrix $M_{a}$ and matrix $M_{b}$. Thus, we can draw a conclusion that matrix $M_{a}$ and matrix $M_{b}$ are similar matrices. In Section 6, we will evaluate the algorithm from both efficiency and accuracy viewpoints.

##### *(b)* *Calculate the Similarity by the Unit of Lines (SUL) Method*

Next, we introduce another algorithm to determine whether two matrices are similar when their line sequences are not certain. We first introduce a formula to calculate the similarity of two rows in two matrices.

(5) $$W\left(ra_{m},rb_{n}\right)=\frac{\sum_{j=1}^{|M_{a}^{c}|}H\left(ra_{\left(m,j\right)},rb_{\left(n,j\right)}\right)}{|M_{a}^{c}|}$$

where $ra_{m}$ is a row of matrix $M_{a}$ and $rb_{n}$ is a row of matrix $M_{b}$. $|M_{a}^{c}|$ is the number of columns of matrix $M_{a}$. $H(ra_{\left(m,j\right)},rb_{\left(n,j\right)})$ is a formula for calculating the similarity of the similarity of matrix element between $a_{\left(m,j\right)}$ and $b_{\left(n,j\right)}$ as follows.

(6) $$H\left(ra_{\left(m,j\right)},rb_{\left(n,j\right)}\right)=\left\{\begin{array}{c} 1,\mathrm{if}\;|a_{\left(m,j\right)}-b_{\left(n,j\right)}|\;<m\\ 0,\mathrm{otherwise} \end{array}\right.$$

If the $W(ra_{m},rb_{n})>s$ (similarity threshold), we put the numbers of these two lines $\left(Row_{a},Row_{b}\right)$ as a line number group in a set (Temp_list), where $Row_{a}$ is the number of $ra_{m}$ and $Row_{b}$ is the number of $rb_{n}$.

We then determine whether the two matrices are similar by analyzing the set of the line number group. We make a two-step moving operation on the set in which the line number group is stored.

- Step 1. Find the line number groups in which $Row_{b}$ is not repeated in the set and move these line number groups to another set (Result_list) for storage.
- Step 2. After performing the moving operation in the first step, if the set Temp_list is not empty, we perform the second moving operation and successively determine the line number groups remaining in the set Temp_list. If the $Row_{a}$ and $Row_{b}$ in this line number group are different from those in the set Result_list, we move this type of line number group out to Result_list, as well.
- Step 3. We determine the number of line number groups in the set Result_list. If the number equals the number of lines in the matrix, it is believed that the two matrices are similar.

**Figure 6:**
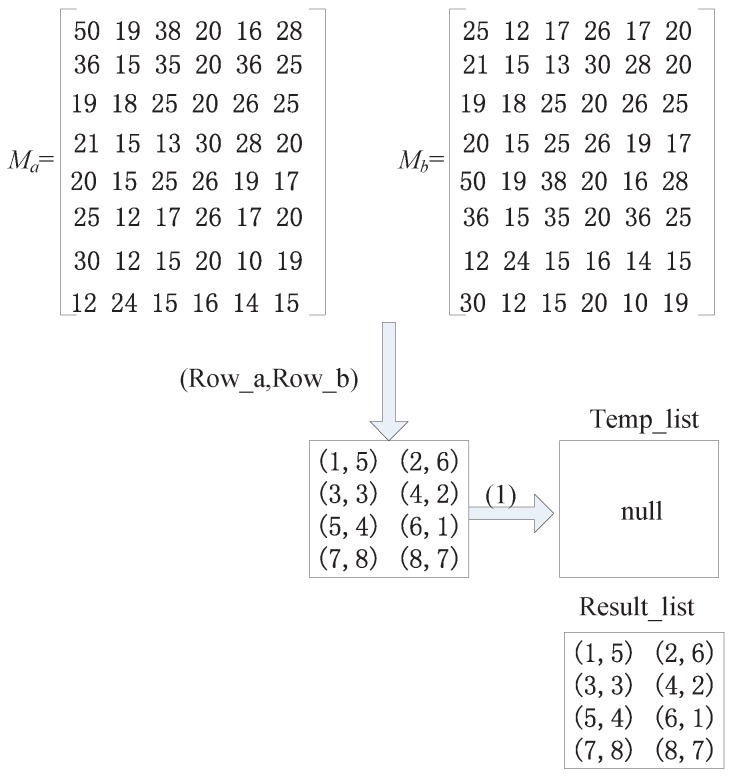
Example 1 for the Similarity by the Unit of Lines (SUL) method.

Here is an example as shown in Figure 6. Matrices $M_{a}$ and $M_{b}$ are matched successively on the basis of the lines. We put the similar line number groups (1, 5), (2, 6), (3, 3), (4, 2), (5, 4), (6, 1), (7, 8) and (8, 7) into the set Temp_list. Then, we perform the first operation on the line number groups in Temp_list. Because the $Row_{b}$ values of these groups are 5, 6, 3, 2, 4, 1, 8, 7 and are not repeated, we can directly move these groups out to Result_list. At this time Temp_list is empty, and we do not have to perform the second step. The number of the line number groups in Result_list is eight and is determined to be the same as the number of lines in matrices $M_{a}$ and $M_{b}$; therefore, matrix $M_{a}$ and matrix $M_{b}$ are similar matrices.

**Figure 7:**
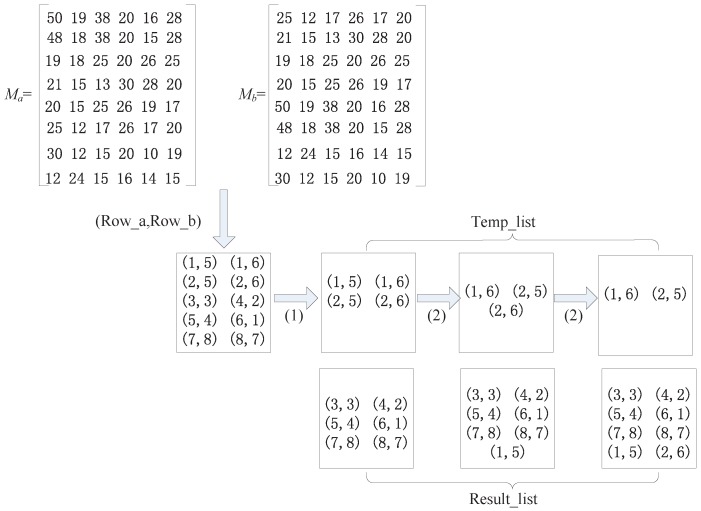
Example 2 for the SUL method.

In the above example, the similarity of lines in matrix $M_{a}$ and matrix $M_{b}$ is in correspondence, but sometimes, the similarity of the lines of two matrices is not in correspondence. As shown in Figure 7, the first line of matrix $M_{a}$ is similar to both the first line and the third line of matrix $M_{b}$. Therefore, calculate the similarity of $M_{a}$ and matrix $M_{b}$ by the unit of lines. Line number groups (1, 5), (1, 6), (2, 5), (2, 6), (3, 3), (4, 2), (5, 4), (6, 1), (7, 8) and (8, 7) are included in Temp_list. Then, we perform the first-step operation on the line number groups in Temp_list. Because the $Row_{b}$ values of these ten line number groups are 5,6,5,6,3,2,4,1,8 and 7 and $Row_{b}=$ 3,2,4,1,8 and 7 are not repeated, we can move (3, 3), (4, 2), (5, 4), (6, 1), (7, 8), (8, 7) out to Result_list. Because at this time Temp_list is not empty, we need to perform the second step to move (1, 5) and (2, 6) to Result_list. After the implementation of the above two steps, we determine that the number of the line number groups in Result_list is eight and is the same as the numbers of lines in matrix $M_{a}$ and matrix $M_{b}$; therefore, matrices $M_{a}$ and $M_{b}$ are similar.

### 4.5. Step 5: Node Location Identification

#### 4.5.1. Localization of Key Nodes

We can identify each intersection node group near a traffic light by considering similar matrices. For example, if the $D_{n}$ (a detection matrix) successfully matches with $K_{m}$ (a known matrix), we can consider the intersection node group ($D_{n}$ is constructed by this intersection node group) to be near this traffic light ($K_{m}$ is constructed by this traffic light information). From what has been discussed above, we can then locate the intersection node group. Because the intersection node group is composed of key nodes, we can locate key nodes.

#### 4.5.2. Localization of Common Nodes

There is an algorithm to use vehicle detection time stamps to locate sensors called the Autonomous Passive Localization (APL) algorithm [21]. This algorithm can obtain distance estimates between any pair of sensors on roadways to construct a virtual graph composed of sensor identifications and distance estimates, so we can identify where common sensors are located on roadways and obtain distance estimates and key nodes. Thus, common nodes can be located because the location of key nodes is known by Section 4.5.1.

## 5. Practical Discussion

### 5.1. Dealing with the Same Traffic Light Schedule

In practice, the possibility that the setting modes of different intersection traffic lights are the same may lead to the same or similar matrices in the matrix collection to make the matrix matching result not unique. Consequently, we cannot locate some intersection node groups. We adopt the following two methods to solve these special location problems of intersection node groups.

- Method 1. There are not two identical matrices in the detection matrix collection, but a matrix in the detection matrix collection has more than one matching result with the matrix of the known matrix collection. Because our detection range is within a region, we can locate these special points according to the surrounding intersection node group. When constructing the known matrix collection, we assign to each matrix of a set its surrounding information. Then, we put the matrix around the matrix of the special group in the detection matrix collection to match each other. If the surrounding information in the detection matrix collection has a successful match, we can obtain the corresponding matching result of the special matrix according to the matrix information around the node.
- Method 2. When the nearby traffic lights have a similar setting or the detection matrix collection has identical matrices, the above method does not work well. Therefore, we also can use the map matching method of the APL algorithm to deal with the same traffic light schedule problem.

In all, the first method can efficiently and quickly handle the case of multiple traffic lights with the same setting. However, it does not work when the nearby traffic lights also have similar settings. Therefore, we provide the second method, which needs some time to match the graph. It can work well when the nearby traffic lights will have similar settings.

### 5.2. The Known Matrix Collection Is Very Large

In some cases, the number of traffic lights is very large, causing the known matrix collection to be very large. Therefore, the efficiency of the algorithm described in Section 4 will be very low and may even fail to work. To alleviate this problem, we propose a new algorithm called the Recursion Matching method (RM). Because we know the intersection information around the intersection, when we construct the known matrix collection, we can also record the surrounding intersection ID in an array. Thus, the matrix of the known matrix collection can constitute a graph in which each vertex is an 8 × 6 matrix. We can reduce the program’s running time by the following steps.

- Step 1. We determine the similarity matrix of the first detection matrix ($D_{0}$) in the known matrix collection. The similarity matrix is considered as the initial matrix.
- Step 2. Once the surrounding matrix of the initial matrix is known, we determine the similarity matrix of the surrounding matrix in detection collection. If we determine the similar matrix of a given surrounding matrix, we consider this surrounding matrix as the initial matrix. Then, we perform Step 2 again. It is important to note, if all of the surrounding matrices of the initial matrix do not match, we will need to choose a new initial matrix. We repeat until all of the matrices of the detection matrix collection are matched successfully.

**Figure 8:**
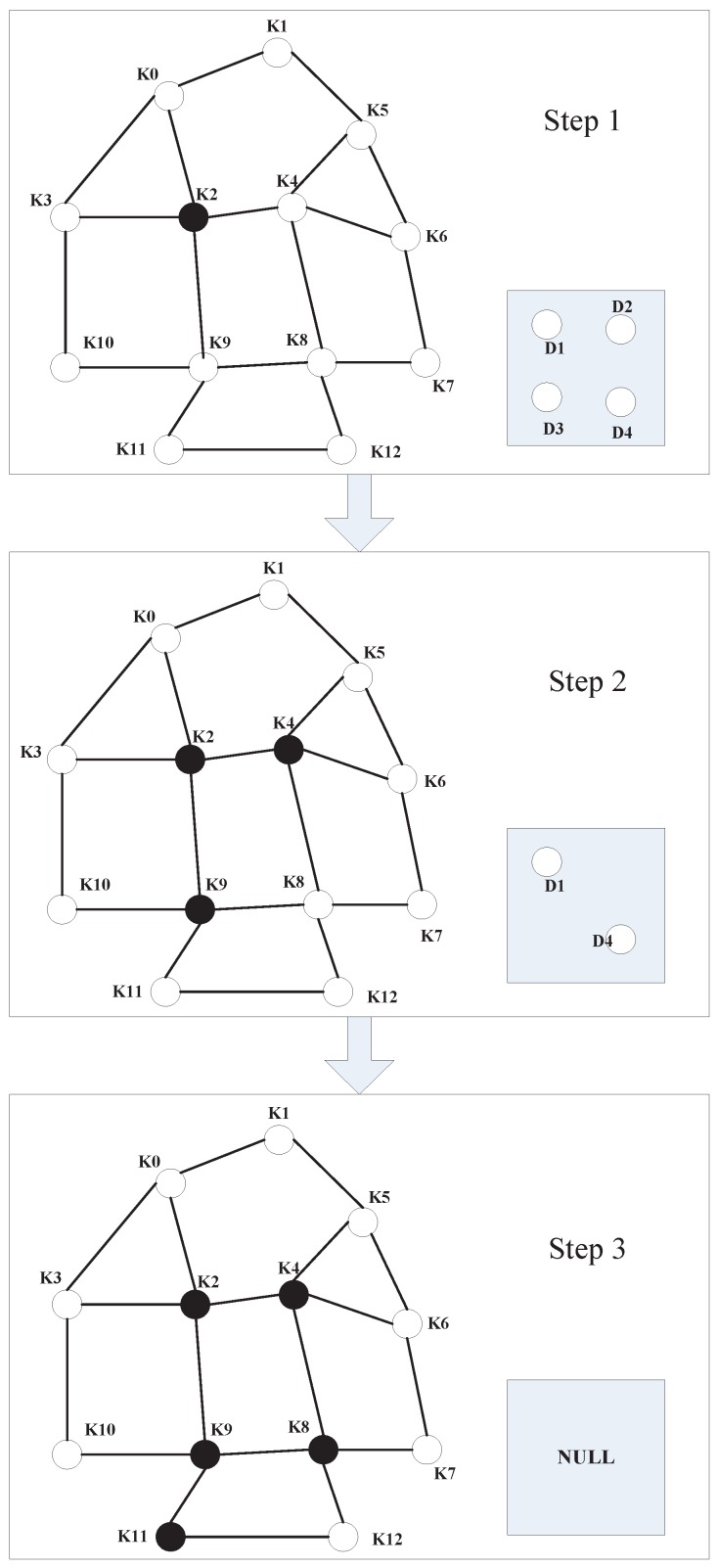
Example of the Recursion Matching (RM) method.

Through the above steps, we can see that the number of matrices in the known matrix collection has only a small effect on the program running time. For example, as shown in Figure 8, in the first step, we find that $D_{0}$ of the detection matrix collection and $K_{2}$ of the known matrix collection are similar matrices. The second step will be $K_{2}$ as a starting point, and then, its surrounding nodes $K_{0},\;K_{3},\;K_{4},\;K_{9}$, in turn, match in the detection matrix collection. $K_{4},\;K_{9}$ can be matched with $D_{2}$, $D_{3}$. Then, recursively match $K_{4}$ and $K_{9}$ with the surrounding vertex until all of the matrices of the detection matrix collection are matched successfully.

### 5.3. Some Key Nodes Damaged

In practice, some key sensors may appear damaged; in other words, the sensor number of some intersection node groups is less than eight. Therefore, some detection matrices have less than eight rows (i.e., 7×6 or 6×6 matrix). In this case, use the SUL method described in Section 4 to judge whether the matrix is similar. Thus, in the case of a small amount of damage to sensors, the TLS algorithm can still work well.

### 5.4. Exciting Some Adaptive Traffic Light Controls

Most intersections have a fixed time scheduling based on a predefined time-plan. It is suitable for managing stable and regular traffic flows, but it may not be able to efficiently cope with dynamically varying traffic conditions. Thus, some traffic lights schedules are not static and are dynamically determined [12]. According to this problem, we can dynamic update the known matrix collection based on the real-time time schedules. Additionally, the newest traffic law can also be collected and matched with the updated known matrix collection.

## 6. Performance Evaluation

In this section, we investigate system parameters and present two types of performance evaluations as follows. First, we study two thresholds (matrix element gap threshold *m* and matrix similarity threshold *s*) in Section 6.1. Second, we compare the RSS method with the SUL method and compare the RM method with the SUL method for accuracy and elapsed time in Section 6.2. Finally, we compare our method with the APL algorithm in Section 6.3 and evaluate the impact of practical factors in Section 6.4.

Define two performance metrics as follows: First, for the matching accuracy comparison, the ratio of the number of incorrectly localized sensors to the number of all sensors is defined as the key nodes’ accuracy ratio. Second, for the matching efficiency comparison, the program running time is defined as elapsed time.

### 6.1. Investigation on System Parameters

In our system, we use two parameters (*m* and *s*) to calculate the similarity of the matrix. To find the best value, we use a C++ language simulator and consider the scenario that 400 sensor nodes are placed at 50 intersections.

**Figure 9:**
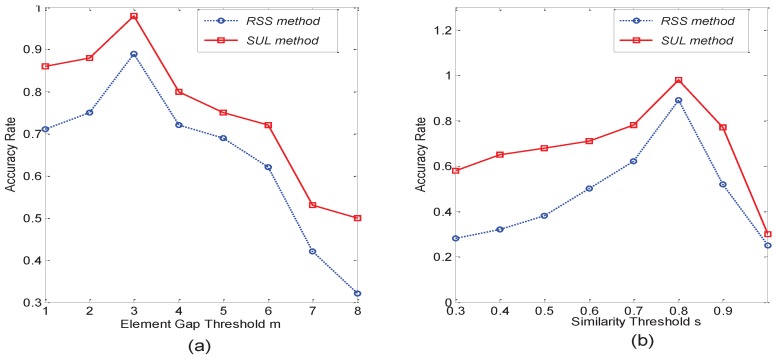
Investigation on system parameters. (**a**) For element gap threshold *m*; (**b**) for similarity threshold *s*.

For the matrix element gap threshold *m*, in Figure 9a, we can see that the key nodes’ accuracy rate of the RSS method and the SUL method is the best when the m=3. For the matrix similarity threshold *s*, in Figure 9b, we can see that the key nodes accuracy rate of RSS method and SUL method is the best when s=0.8. Thus, the best choice is m=3 and s=0.8 for our system.

### 6.2. Performance Comparison between Matrix Matching Methods

We compare the performance of matrix matching methods according to the following three methods:

- Row Sum Sorting method (RSS)
- Calculate the Similarity by the Unit of Lines method (SUL)
- Recursion Matching method (RM)

**Figure 10:**
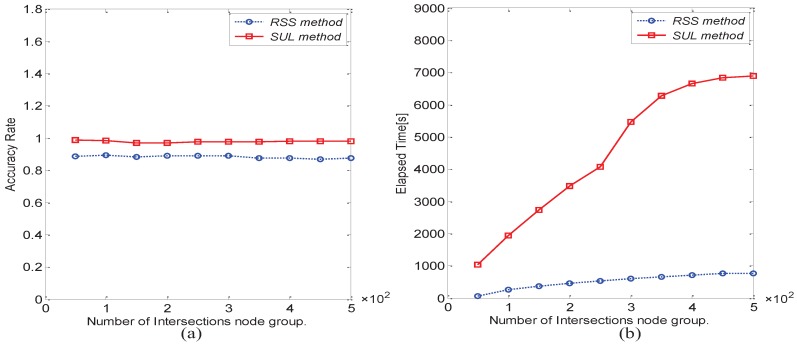
Comparison between the RSS method and the SUL method. (**a**) For key nodes accuracy rate; (**b**) For elapsed time.

**Figure 11:**
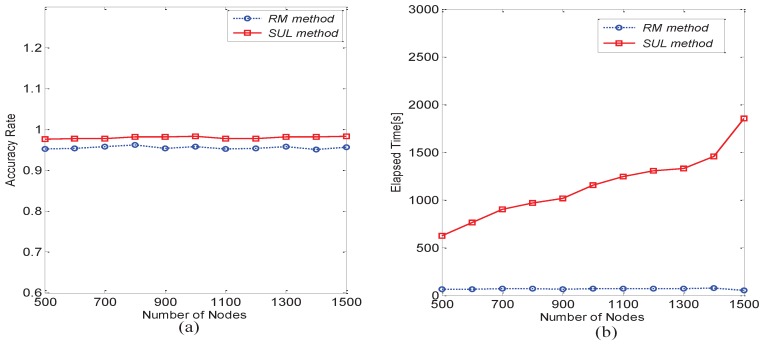
Comparison between the RM method and the SUL method. (**a**) For key nodes’ accuracy rate; (**b**) For elapsed time.

For the accuracy comparison of the RSS and SUL methods, as shown in Figure 10a, the key nodes’ accuracy ratios of the RSS and SUL methods are relatively high, and the SUL method is higher than the RSS method. As shown in Figure 10b, we find that the RSS method uses less elapsed time than the SUL method; therefore, the RSS method has a better efficiency. Figure 11a,b compares the RM method with the SUL method when the known matrix collection is very large. We also find that their accuracy is essentially the same. With the increase of the number of known matrices, the elapsed time of the SUL method rises quickly, but the elapsed time of the RM method remains basically unchanged. Thus, the RM method is more suitable for the case in which the known matrix collection is very large.

### 6.3. Performance Comparison between the TLS Algorithm and the APL Algorithm

**Figure 12:**
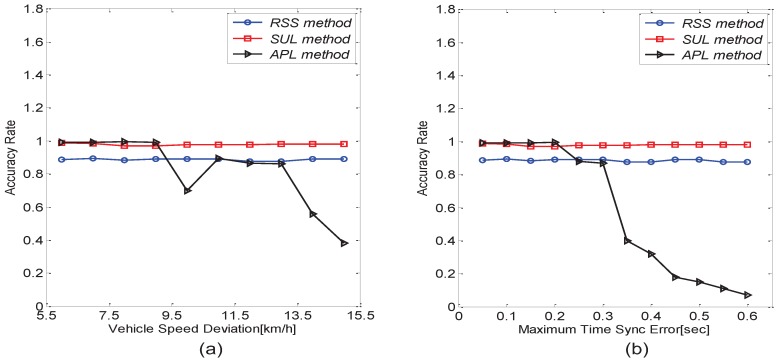
Comparison between the Traffic Lights Schedule (TLS) algorithm and the Autonomous Passive Localization (APL) algorithm. (**a**) The impact of vehicle speed deviation; (**b**) the impact of maximum time sync error.

The TLS algorithm and the APL algorithm both use only vehicle detection time stamps to locate sensors; therefore, we compare the two algorithms. The common nodes that are located by map matching have the same accuracy rate of the APL algorithm, so we focus on the accuracy rate of key nodes. We find that the TLS algorithm has a wider range of application than the APL algorithm because the APL algorithm is affected by time synchronization error and vehicle speed deviation. As shown in Figure 12a, we can see that the APL algorithm works well in the case in which the vehicle standard deviation is less than 10 km/h, but the TLS algorithm is not affected by vehicle speed deviation. As shown in Figure 12b, it can be seen that the APL algorithm works well in the case in which the maximum time synchronization error is less than 0.25 s. However, our TLS algorithm is not affected by maximum time synchronization error. In summary, the TLS algorithm has a wider range of application compared with the APL algorithm.

### 6.4. Impact of Practical Factors

**Figure 13:**
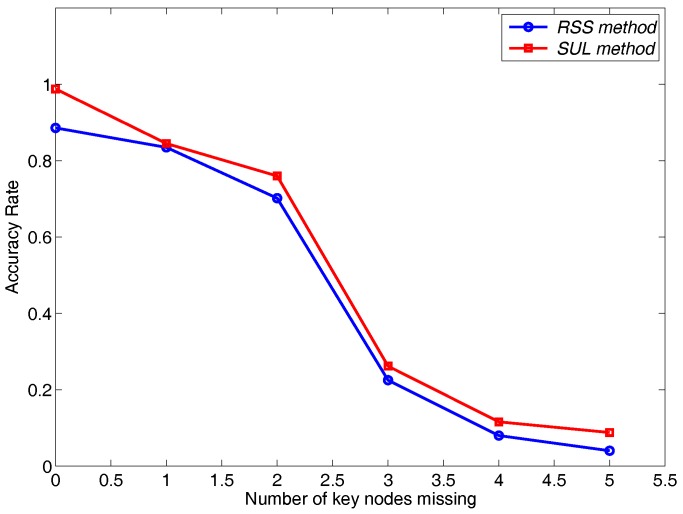
The impact of missing key nodes.

**Figure 14:**
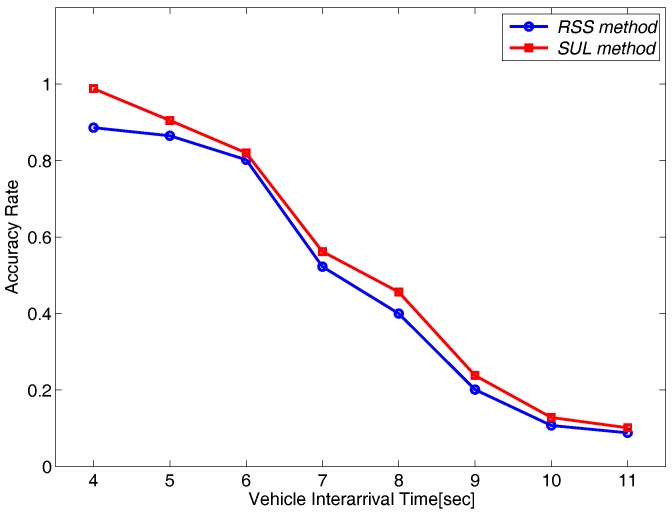
The impact of heavy traffic.

We evaluate the impact of heavy traffic and the number of missing key nodes in an intersection. Our localization scheme works well in the case that the traffic is heavy and there are no missing key nodes. As shown in Figure 13, with the increasing of the missing key nodes, the performance of TLS is a downward trend. The average vehicle inter-arrival time can also reflect the condition of traffic. Hence, we give the accuracy change trend with the increasing of vehicle inter-arrival time. As shown in Figure 14, we see that the traffic can affect the performance of the localization scheme. That is because sparse traffic offers low information to localize the nodes.

## 7. Conclusions

In sparse urban road networks, because sensors cannot effectively obtain pairwise ranging distance or connectivity information, the previous localization schemes do not work well. Unlike previous localization schemes, this work introduces a novel localization scheme, called TLS, using binary sensors and traffic light schedule information. Our TLS system performs a localization using vehicle detection time stamps along with the traffic lights information of the target area. The key idea is to use the statistics of vehicle detection time stamps to obtain the traffic lights law of each intersection to construct a detection matrix collection, which is then matched with the known matrix collection, to identify where sensors are located in the target road network. For calculating the similarity of two matrices, we can judge the similarity of two matrices even if the row order of a matrix is uncertain. Finally, our algorithm is evaluated by extensive simulation. The results show that the localization accuracy of intersection sensors can reach more than 90%. In addition, we compare with existing typical algorithms and find that the TLS algorithm has a wider operational region.

Although the TLS algorithm can provide a good performance, there is room for further enhancements. TLS algorithm has some limitations. For example, the data of vehicle detection time must include heavy traffic time. In future work, we will try to apply other public facilities information (e.g., public buses) to improve our localization algorithm, in order to make our algorithm work well when the vehicle traffic is sparse.

## Figures and Tables

**Table 1:** Simulation configuration.

Parameter	Description
number of vehicle arrivals within an hour *λ*	λ=600 represents 600 vehicles arrival within an hour.
Time segment *t*	t=1/6 represents a time segment of 10 min.
number of vehicle arrivals within time segment *n*	n=200 represents 200 vehicles arriving within 10 min.
determining valid data error probability $P_{error}$	It represents the probability of n>200 in the sparse time segment.

